# Reflecting on tuberculosis case notification and treatment outcomes in the Volta region of Ghana: a retrospective pool analysis of a multicentre cohort from 2013 to 2017

**DOI:** 10.1186/s41256-019-0128-9

**Published:** 2019-12-17

**Authors:** Eric Osei, Samuel Oppong, Daniel Adanfo, Bless Ativor Doepe, Andrews Owusu, Augustine Goma Kupour, Joyce Der

**Affiliations:** 1 Department of Population and Behavioural Sciences, School of Public Health, University of Health and Allied Sciences, Ho, Ghana; 2 Department of Epidemiology, School of Public Health, University of Health and Allied Sciences, Ho, Ghana

**Keywords:** Tuberculosis, Case notification, Treatment outcomes, Volta region, Ghana

## Abstract

**Background:**

Tuberculosis (TB) remains a petrified condition with a huge economic and health impact on families and health systems in Ghana. Monitoring of TB programme performance indicators can provide reliable data for direct measurement of TB incidence and mortality. This study reflects on the trends of TB case notification and treatment outcomes and makes comparison among 10 districts of the Volta region of Ghana.

**Methods:**

This was a retrospective analysis of surveillance data of a cohort of TB cases from 2013 to 2017. Trends of case notification and treatment outcomes were examined and compared. Logistic regression was used to determine the independent relationship between patients and disease characteristics and unsuccessful treatment outcomes. Odds ratios, 95% confidence intervals and *p*-values were estimated.

**Results:**

A gradual declining trend of case notification of all forms of TB was noticed, with an overall case notification rate (CNR) of 65 cases per 100,000 population during the period. A wide variation of case notification of TB was observed among the districts, ranging from 32 to 124 cases per 100,000 population. Similarly, treatment success rate decreased slightly from 83.1% during the first year to 80.2% in 2017, with an overall treatment success rate of 82.5% (95% CI: 81.3–83.8%). Treatment failure, death, and lost to follow up rates were 0.8% (range 0.5–1.2%), 13.5% (range 12.4–14.7%), and 3.1% (range 2.6–3.8%) respectively. The treatment success rate among districts ranged from 70.5% in South Tongu to 90.8% in Krachi West district. Returned after treatment interruption (Adjusted odds ratio [AOR]: 3.62; 95% CI: 1.66–7.91; *P* < 0.001) and TB/HIV co-infection (AOR: 1.94; 95% CI: 1.57–2.40; *P* < 0.001) predicts poor treatment outcomes.

**Conclusion:**

Over the past five years, TB case notification and successful treatment outcomes did not significantly improve. Wide district variations in CNR was observed. The overall treatment success rate observed in this study is below the target of > 90% set by the World Health Organization’s (WHO) end TB strategy. Additionally, patients who returned to continue treatment after interruption and those who were co-infected with HIV strongly predict unsuccessful treatment outcomes. Sustained interventions to prevent treatment interruptions and improved management of co-morbidities can enhance treatment outcomes, as required to achieve the elimination goal.

## Background

Despite the availability of effective drug treatment since the 1940s, coupled with social and economic development, Tuberculosis (TB) remains a major public health problem globally, with millions of people affected by the disease each year. Geographically, the burden of TB is highest in Asia and the Africa regions, with Africa accounting for about 25% of global TB incident cases [1]. Globally, about 10 million people were estimated to have developed TB disease in 2017, however, only 6.4 million (64%) were notified to national authorities and then to the World Health Organization (WHO). The shortfall in numbers could be due to underreporting of diagnosed cases or people not being diagnosed when they seek care, or they do not seek care at all [2]..

The major goals of Tuberculosis treatment are to cure the individual with the disease, reduce death and disability, and minimize the spread of *Mycobacterium tuberculosis* to other persons in the community. Nonadherence to TB treatment could lead to treatment failure, relapse, development of drug resistance, and prolonged infectiousness of patients [3]. Cognizant of this fact, the WHO introduced Directly Observed Treatment Short-course (DOTS) as a standard strategy for TB control since 1993. The aim of the strategy is for all National TB control programmes to detect at least 70% of estimated infectious cases and successfully treat 85% of them [4].

In Ghana, TB has remained a petrified condition for many years with a huge economic and health impact on individuals as well as the health system in general [5]. In 2018, 13,978 incident cases of TB, representing 32% of expected cases were detected in the country and notified to WHO [2] Recent national TB prevalence survey in 2013 estimated a prevalence of 264 cases per 100,000 population for all forms of TB and bacteriological prevalence of 356 per 100,000 population with smear-positive rate of 105 per 100,000 population [6], indicating that TB is still a major public health challenge in Ghana.

Standardized monitoring of notifications of TB cases and their treatment outcomes has been ongoing since 1995. In the era of the End TB Strategy and Sustainable Development Goals (SDGs), such monitoring needs to be sustained, together with unrelenting efforts to improve case notification in order to provide reliable data for direct measurement of TB incidence and mortality [2]. Additionally, such analysis enables National Tuberculosis Programmes to monitor trends in the occurrence and distribution of TB cases across a geographical location [1]. Few studies in Ghana, have reported TB treatment outcomes. However, none of these studies made a comparison of districts. Tetteh and colleagues, for instance, used data from a referral hospital [7], while Amo-Adjei et al. [5] used national data to make regional comparison. Furthermore, to the best of our literature search, no study has assessed the trends of TB case notification in Ghana. This study, therefore, reflects on the trends of TB case notification and treatment outcomes from 2013 to 2017 and made comparison among 10 districts in the Volta region of Ghana.

## Materials and methods

### Study settings and design

We conducted a retrospective cross-sectional study of all TB cases registered from 2013 to 2017 in 10 TB Basic Management Units (BMU)/ districts of the Volta Region of Ghana. A BMU is defined in terms of management, supervision, and monitoring responsibilities. Each unit had one or more treatment centres, laboratories, and a hospital. There is a coordinator who oversees TB control activities for the unit and maintains a master register of all Patients with TB being treated. The Volta Region is bound to the north by the Northern Region, the south by the Gulf of Guinea, west by the Volta Lake and the east by the Republic of Togo. It is divided into three natural geographical belts namely the southern, middle and the northern belts and has 25 administrative districts with 377 health facilities, serving a population of over 2,789,211. There are 44 DOTS centres and 41 laboratories in the region that provide TB diagnostic and treatment services to TB clients. At the DOTS centres, TB diagnosis, treatment, and monitoring are done as per the national tuberculosis control program (NTBCP) guidelines [8]. As in all other parts of Ghana, there are also TB/HIV collaborative activities at all DOTS centres in the region.

### Sampling and study population

For representativeness, stratified sampling was used to select participating districts. Strata were the geographical belts (southern, middle and northern) of the region. Five districts in the region did not have data from 2013, hence were not included in the strata for selection. Ten (50%) districts were then selected from the remaining twenty. We randomly selected four districts each from the South (Keta, Ketu South, Central Tongu, and South Tongu) and Northern (Nkwanta South, Krachi West, Kadjebi, and Krachi East); and two from the middle belt (Kpando and Hohoe) using probability proportion to the number of districts in each stratum. Study population constituted patients with all forms of TB who were registered and commenced anti-TB treatment between 1st January 2013 and 31st December 2017 in the participating districts.

### Data source and extraction

Data were extracted from the master registers of each BMU. The registers are provided by the Ghana NTBCP and are used by all districts in Ghana. The registers are used to record information of all Patients with TB been treated at all DOTS centres in the districts, which is used to monitor the programme and report on indicators to the higher level. Information contained in the register include patients’ demographics; type and category of TB; initial and follow up sputum smear examination results; treatment outcomes; and TB/HIV collaborative activities. Data were extracted directly into Microsoft Excel spreadsheet designed to capture all relevant variables. Data extractions were done by the researchers in the presence of District health staffs. Variables such as age, sex, types of TB, co-morbidity with HIV/AIDS, and treatment outcomes were extracted for analysis. To ensure data quality and accuracy, the district TB focal persons were made to systematically check and compare extracted data with the source document. Data were cleaned to check for consistency and completeness of the data set before exporting for analysis.

### Outcome measures and definitions

We assessed the outcome measures of TB treatment in this study as percentages of successful and unsuccessful outcomes among all patients who started treatment. Successful treatment outcomes included cured patients and those who completed treatment. Unsuccessful treatment outcomes included failures, lost to follow-ups and deaths. We excluded transferred out and those whose outcomes were missing in our analysis of treatment outcomes. We used WHO’s definitions for surveillance of Patients with TB before and after treatment [9].

### Data handling and analysis

Data was exported into Stata Version 13.1 (Stata Corp, College Station, Texas, USA) for analysis. Data were summarized using frequencies and proportions to describe the study population in relation to relevant variables. Case Notification Rate (CNR) was computed as the total number of cases notified divided by the estimated annual population and was expressed in 100,000. We used the Chi-square test to compare two proportions and applied trend tests across the 5 years to test for changes in CNR and treatment outcome trends. Bivariate and multivariate logistic regression were used to identify significant predictors of unsuccessful treatment outcomes, which was the main outcome variable, defined as patients who died, were lost to follow up or failed treatment. First, the association of unsuccessful treatment outcomes with each variable of interest was examined, ignoring all other variables. Second, variables with *p*-value < 0.05 were considered for inclusion to construct a model with risk factors independently associated with unsuccessful treatment outcomes. Patients’ age, sex, category and type of TB, TB/HIV co-morbidity, district, and year of treatment were included as covariates. The degree of association between dependent and independent variables was assessed using odds ratios (OR) with 95% confidence intervals (CI). To test for the goodness of fit for the model, we used the likelihood ratio test to compare the likelihood of the data under the full model against the likelihood of the data under a model with reduced independent variables and since the *p*-value for the overall model fit statistic was less than 0.05, we concluded that the model is good.

### Ethical statement

Ethical approval was obtained from the University of Health and Allied Sciences ethical review committee. Informed consent was not obtained since there was no direct contact with patients. Patients’ identifying information were not collected from the registers. Permission was sought from the Volta regional health Director and District Directors of participating districts before data were extracted. Data extractions were done in the presence of district health staff.

## Results

### General characteristics of study subjects

Over the 5 years, 3735 TB cases of all forms were reported in the study districts, of whom ages ranged between 1 and 96 years, with median (interquartile range) age of 44 (19–69) years. Children under 15 years accounted for 4.3% (161) of all reported cases, while 15.4% (574) were elderly (64+ years). Majority (2335; 62.5%) were males, most 3578 (95.8%) were registered as new cases, while 138 (3.7%) were retreatment cases, among which 55 (1.5%) were relapse, 33 (0.8%) were returned after default, and 25 (0.7%) were treatment failure cases. Of all reported cases, 1712 (45.8%) were pulmonary bacteriologically confirmed, 1900 (50.9%) were smear-negative cases, while 116 (3.1%) were extrapulmonary TB cases. Regarding HIV status, 712 (19.1%) of all cases were HIV positive, while HIV status of 591 (15.8%) was unknown. Majority (1125; 30.1%) of cases were from Ketu South District, followed by Keta and Hohoe (11.8 and 11%), and the least (123; 3.3%) from Krachi West, followed by from Krachi East (189; 5.1%) and Central Tongu (197; 5.3%) (Table 1).

**Table 1:** General characteristics of Patients with TB in 10 districts of Volta region, Ghana (2013–2017)

Characteristics	Male (*N* = 2335) *n*(%)	Female (*N* = 1400) *n*(%)	Total (*N* = 3735) *n*(%)
*Age group (yeas)*			
< 15	80 (49.7)	81 (50.3)	161 (4.3)
15–24	152 (48.9)	159 (51.1)	311 (8.3)
25–34	339 (56.4)	262 (43.6)	601 (16.1)
35–44	555 (65.1)	297 (34.9)	852 (22.8)
45–54	501 (67.4)	242 (32.6)	743 (19.9)
55–64	343 (69.5)	150 (30.4)	493 (13.2)
65+	365 (63.59)	209 (36.4)	574 (15.4)
*Patient category*			
New	2225 (62.2)	1353 (37.8)	3578 (95.8)
Retreatment*	98 (71%)	40 (29%)	138 (3.7)
Transferred in	12 (63.2)	7 (36.8)	19 (0.5)
*TB classification*			
Smear positive Pulmonary ^¶^	1120 (65.5)	591 (34.5)	1711 (45.8)
Smear negative Pulmonary^§^	1147 (60.43)	753 (39.6)	1900 (50.9)
Extra pulmonary	64 (55.2)	52 (44.8)	116 (3.1)
Missing −1	3 (42.9)	4 (57.1)	7 (0.2)
*HIV status*			
Positive	353 (49.6)	359 (50.4)	712 (19.1)
Negative	1619 (66.6)	813 (33.4)	2432 (65.1)
Not known	363 (61.4)	228 (38.6)	591 (15.8)
*District of registration*			
Central Tongu	120 (60.9)	77 (39.1)	197 (5.3)
Hohoe	300 (72.8)	112 (27.2)	412 (11.0)
Kadjebi	224 (68.3)	104 (31.7)	328 (8.8)
Keta	280 (63.6)	160 (36.4)	440 (11.8)
Ketu South	661 (58.8)	464 (41.2)	1125 (30.1)
Kpando	238 (64.9)	129 (35.1)	367 (9.8)
Krachi East	100 (52.9)	89 (47.1)	189 (5.1)
Krachi West	78 (63.4)	45 (36.4)	123 (3.3)
Nkwanta South	208 (60.7)	136 (39.3)	344 (9.2)
South Tongu	126 (60.0)	84 (40.0)	210 (5.6)
All districts	2335 (62.5)	1400 (37.5)	3735 (100)

*include returned after default (33), relapse (55), failure (25), Multi-drug resistant (1) and other previously treated cases whose previous treatment outcome was unknown (24); ^¶^Also referred to as Pulmonary bacteriologically confirmed; ^§^Clinically diagnosed

### TB notification and trends

**Fig. 1:**
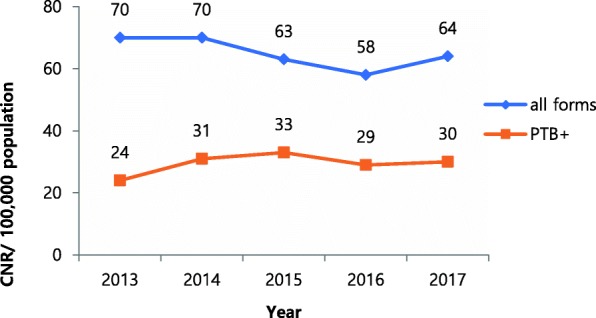
Overall trends of TB case notification rate, 2013–2017

**Fig. 2:**
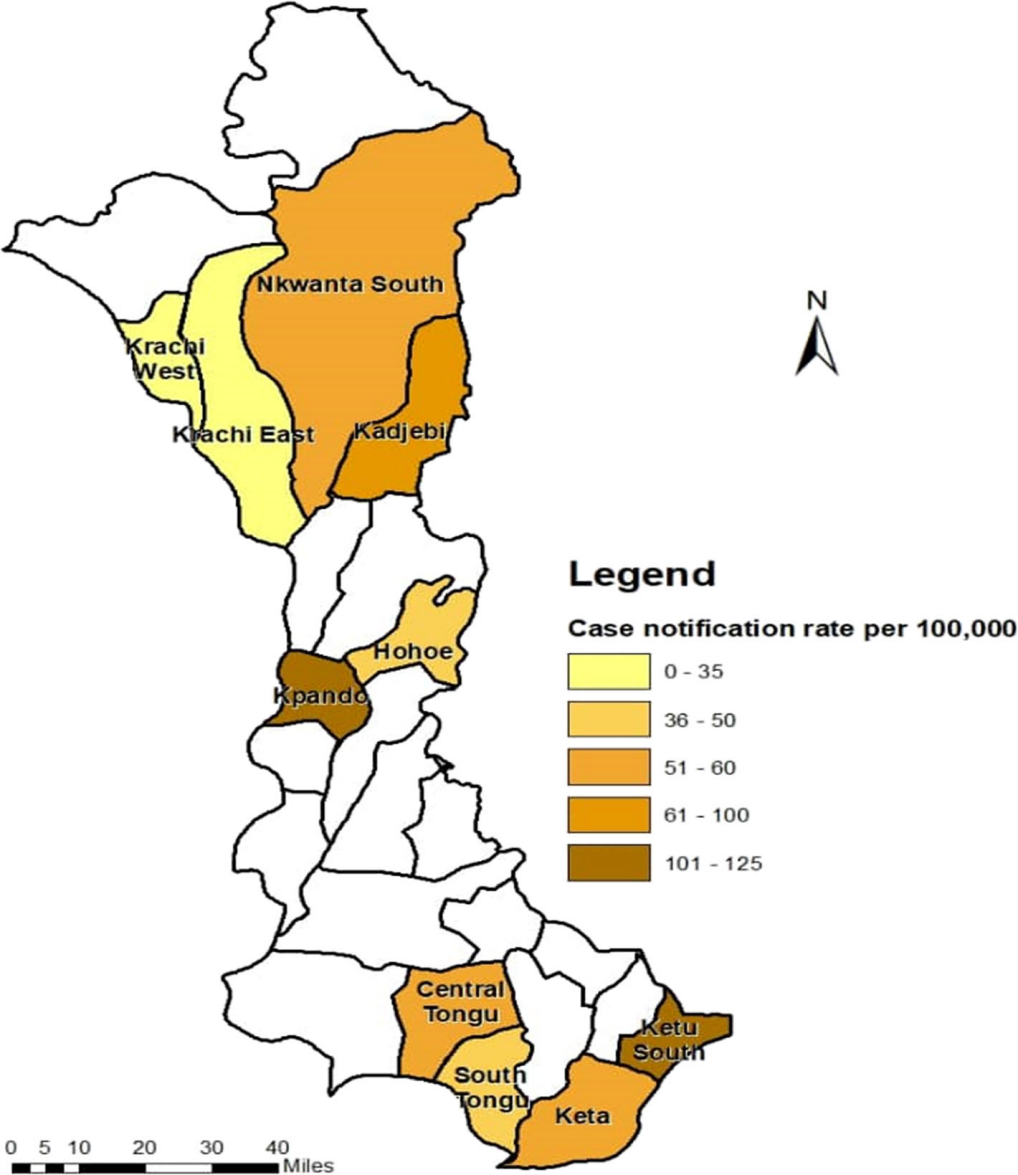
TB Cases Notification Rate (all forms) by District, 2013–2017

During the period 2013–2017, an overall downward trend of 8.6% was observed in the notification of incident TB cases from 70 to 64 cases per 100,000 population (*p* = 0.11). The case notification rate of new pulmonary bacteriologically confirmed cases, on the other hand, rose by 25% from 24 to 30 cases per 100,000 population (*p* = 0.49) (Fig. 1). Five-year case notification rate (CNR) of all forms of TB for the 10 districts was 65 per 100,000 and that of pulmonary bacteriologically confirmed TB was 30 per 100,000 population. The reported rate varied widely among districts, from 124 (Ketu South) to 32 (Krachi East) per 100,000 population. The 5-year CNR of all forms of TB was less than 50 per 100,000 in four districts, between 51 and 100 per 100,000 in another four districts, and higher than 100 per 100,000 in two districts (Fig. 2).

**Fig. 3:**
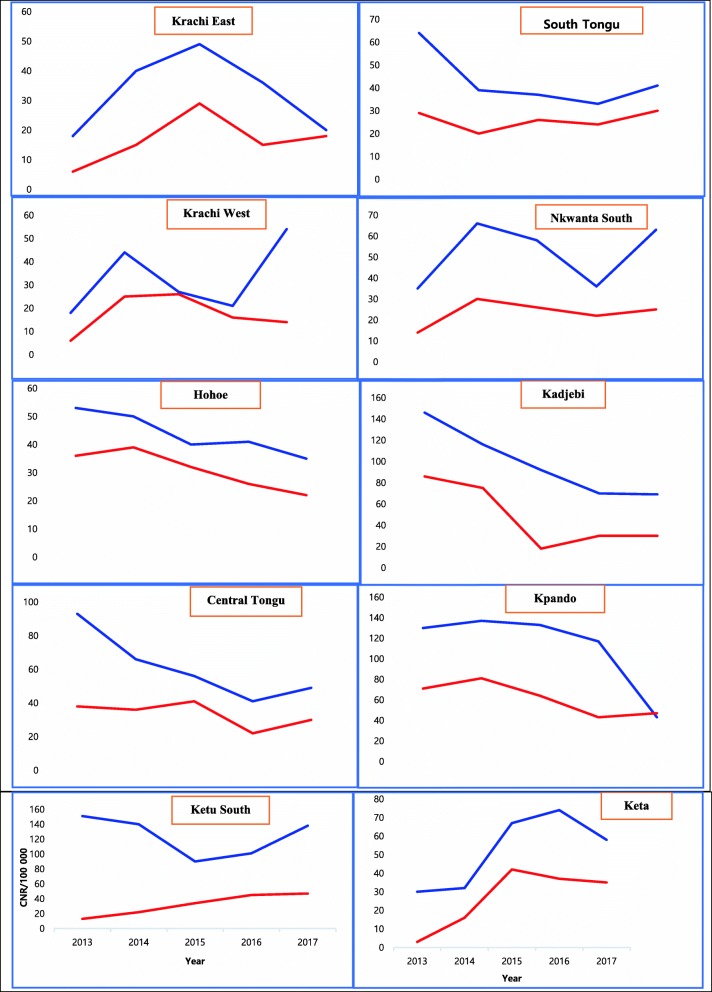
District Trends in TB case Notification Rate, Volta Region, Ghana, 2013–2017. The blue line shows all forms of TB and the red line shows Smear positive pulmonary TB only

Variations in the trends of CNR was observed among districts. Whilst a downward trend in CNR was noticed in Kadjebi, Central Tongu, Hohoe, Kpando, and South Tongu districts, an irregular trend was observed in Krachi East, Krachi West, Ketu South, and Nkwanta. Keta district saw a rise in CNR from 2014 through 2016 and declined thereafter. A similar trend was observed among all new and relapse TB cases and pulmonary bacteriologically confirmed cases (Fig. 3). The biggest decline of CNR of new and relapse TB cases was observed in Kpando (130 to 47 per 100,000) with a decline rate of 64%, followed by Kadjebi (146 to 69) representing 53% reduction, while Krachi West recorded the biggest rise from 18 to 54 per 100,000, representing 200% change, followed by Krachi East (18 to 49) per 100,000 population, with a change of 172%.

### TB treatment outcomes and trends

**Fig. 4:**
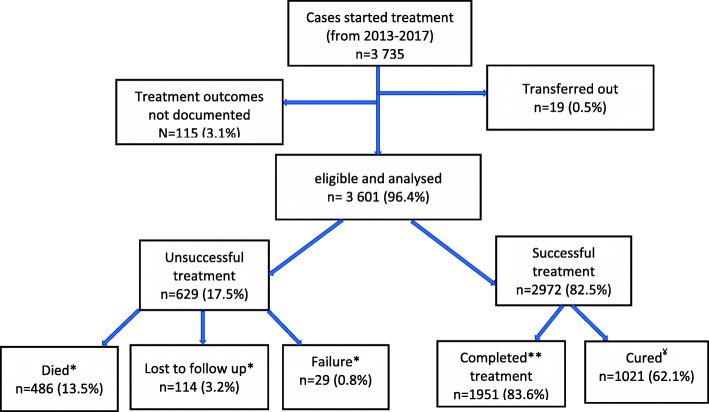
Flow chart of TB treatment outcomes in 10 districts of the Volta Region, Ghana from 2012-2016

**Fig. 5:**
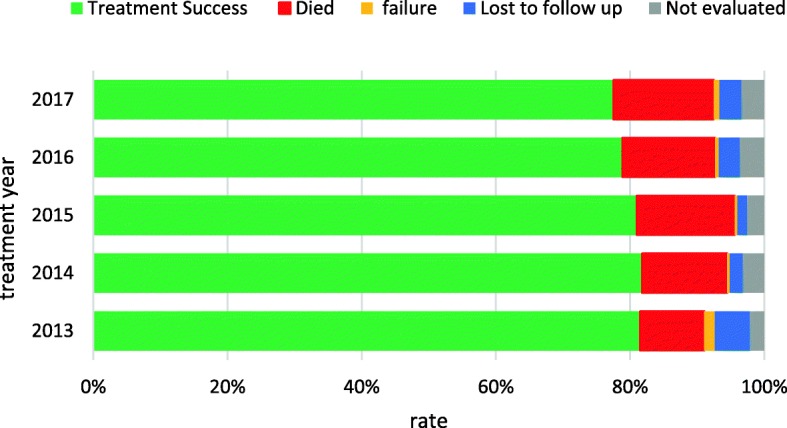
Trends of TB treatment outcomes in 10 districts of the Volta region, Ghana, 2012-2016

Of the 3737 patients treated during the period, treatment outcomes of 3601(94.6%) were evaluated as shown in Fig. 4. Of these, the overall treatment success rate (cured+completed treatment) was 82.5% (95% CI: 81.3–83.8). Of all cohort of TB cases who started treatment, 486 (13.5%) were reported to have died, 3.2% were lost to follow up, and 0.8% had treatment failure. Figure. 5 presents the trends of TB treatment outcomes of 2012 to 2016 cohorts. Treatment success rate decreased slightly from 83.1% (95% CI: 80.1–85.7) in 2013 to 80.2% (95% CI: 77.2–83.0) by December, 2017 (*p* = 0.49). On the other hand, mortality rate rose steadily from 9.8% in 2013 to 15.5% in 2017; *p* = 0.25, while treatment failure rate and lost to follow-ups decreased from 1.6 to 0.9% (*p* = 0.67) and 5.3 to 3.4% (*p* = 0.72) respectively.

### Treatment outcomes by district

Treatment success rate varied among districts with the highest rate observed in Krachi West (90.8%; 95% CI: 84.1–95.3) followed by Kadjebi (86.1%; 95% CI: 81.9–89.7) and Krachi East (85.9%; 95% CI: 79.6–90.6). South Tongu recorded the lowest treatment success rate of 70.5% (95% CI: 63.8–76.6) followed by Central Tongu (71.3%; 95% CI: 64.4–77.5). Treatment failure and death rates were highest in South Tongu (3.9 and 20.7%) and Central Tongu (3.1 and 21.5%) respectively. Keta, Nkwanta South, and Kpando did not record any treatment failure case. Death rate was lowest in Krachi East (3.1%) followed by Krachi West (5.0%). Lost to follow rate ranged from 0% in Keta to 8.5% in Krachi East during the 5 years (Table 2).

**Table 2:** Five-year treatment outcomes of Tuberculosis by district, 2012-2016

Districts	Treatment Success	Unsuccessful			Unknown*	
	Cured + completed	Treatment failure	Died	Lost to follow up	Not documented	Transferred out
	*n*(%) (95% CI)	*n*(%) (95% CI)	*n*(%) (95% CI)	*n*(%) (95% CI)	%	%
Ketu South	885 (81.9) (79.5–84.2)	1 (0.1) (0.02–0.5)	140 (12.9) (11.0–15.1)	54 (5.1) (3.7–6.4)	4.0	0
Keta	374 (85.0) (81.3–88.2)	0	66 (15.0) (11.8–18.7)	0	0	0
South Tongu	146 (70.6) (63.8–76.6)	8 (3.9) (1.7–7.4)	43 (20.7) (15.5–26.9)	10 (4.8) (2.3–8.7)	0	0.1
Nkwanta South	284 (84.8) (80.5–88.4)	0	48 (14.2) (10.8–18.5)	3 (1.0) (0.2–2.5)	2.3	0.6
Hohoe	306 (82.1) (77.8–85.8)	8 (2.1) (0.9–4.2)	57 (15.3) (11.8–19.3)	2 (0.5)(0.07–1.9)	9.2	0.2
Kpando	311 (85.2) (81.1–88.7)	0	48 (13.2) (9.9–17.1)	6 (1.6) (0.6–3.5)	0	1.0
Kadjebi	279 (86.1) (81.9–89.7)	1 (0.3) (0.07–1.7)	31 (9.6) (6.7–13.3)	13 (4.0) (2.2–6.8)	0	1.2
Krachi West	108 (90.7) (84.1–95.3)	1 (1.0) (0.02–4.6)	6 (5.0) (1.8–10.7)	4 (3.3) (1.0–8.3)	0	3.3
Central Tongu	139 (71.3) (64.4–77.5)	6 (3.1) (1.1–6.6)	42 (21.5) (15.9–27.9)	8 (4.1) (1.8–7.8)	0	1.0
Krachi East	140 (85.9) (79.6–90.8)	4 (2.5) (0.7–6.2)	5 (3.1) (1.0–7.0)	14 (8.5) (4.8–13.9)	12.7	1.1
All districts	2972 (82.5) (81.3–83.8)	29 (0.8) (0.5–1.2)	486 (13.5) (12.4–14.7)	114 (3.2) (2.6–3.8)	3.1	0.5

*Not included in estimating treatment outcomes of patients

### Predictors of unsuccessful treatment outcomes

As presented in Table 3, patients who returned to continue treatment after default were more likely to have unsuccessful treatment outcomes (Adjusted OR: 3.62; 95% CI: 1.66–7.91: *P* < 0.001) compared to new cases. The odds of having unsuccessful treatment outcomes was higher among relapse cases (AOR: 1.79; 95% CI: 0.94–3.51; *P* = 0.10) treatment failures (AOR: 1.95; 95% CI: 0.72–5.58; *P* = 0.19) and cases classified as others (AOR: 1.37; 95%CI: 0.48–4.18; *P* = 0.58) compared to new cases, however statistical evidence to support this was weak. TB and HIV co-infected patients were 94% more likely to have unsuccessful treatment outcome (95% CI: 1.57–2.40; *P* < 0.001) compared to TB only patients. Except for South Tongu district, patients treated in all other 8 districts were significantly less likely to have unsuccessful treatment compared to patients treated in Central Tongu District. The proportion of patients with unsuccessful treatment in South Tongu (29.5%) was not statically different from that from Central Tongu (28.7%). Year of treatment, age, sex and patient classification did not predict treatment outcomes.

**Table 3:** Logistic regression of patient and disease characteristics as predictors of unsuccessful treatment outcomes

Variable	Unsuccessful treatment *n* (%)	COR	95% CI	*P*-value	AOR	95% CI	*P*-value
*Age group (years)*				0.37			
< 15	22 (14.4)	1					
15–64	516 (17.9)	1.29	0.82–2.1				
64+	91 (16.3)	1.16	0.69–1.91				
*Sex*				0.33			
Female	225 (16.7)	1					
Male	404 (17.9)	1.09	0.91–1.31				
*Patient category*				0.002			
New	593 (17.1)	1			1		
Default	14 (48.3)	4.52	2.16–9.41		3.62	1.66–7.91	0.001
Relapse	13 (25.5)	1.56	0.88–3.12		1.73	0.89–3.36	0.10
Treatment Failure	5 (22.7)	1.42	0.52–3.87		1.99	0.72–5.58	0.18
Others*	4 (20.0)	1.21	0.40–3.63		1.37	0.45–4.18	0.58
*TB classification*^§^				0.010			
EP	14 (13.1)	1			1		
PP	255 (15.4)	1.21	0.68–2.16		0.96	0.54–1.74	0.90
PN	360 (19.5)	1.61	0.91–2.85		1.29	0.72–2.31	0.40
*HIV status*				< 0.001			
Negative	347 (14.7)	1			1		
Positive	180 (26.5)	2.09	1.71–2.57		1.94	1.57–2.40	< 0.001
Unknown	102 (18.2)	1.29	1.01–1.64		1.17	0.91–1.52	0.22
*District of treatment*				< 0.001			
Central Tongu	56 (28.7)	1			1		
Keta	66 (15.0)	0.44	0.29–0.66		0.49	0.28–0.67	0.001
Hohoe	67 (17.9)	0.54	0.36–0.82		0.61	0.39–0.93	0.02
Ketu South	195 (18.6)	0.55	0.39–0.77		0.51	0.36–0.74	< 0.001
Krachi West	11 (9.2)	0.25	0.13–0.51		0.29	0.15–0.59	0.001
Krachi East	23 (14.1)	0.41	0.24–0.69		0.45	0.26–0.78	0.005
Kpando	54 (14.8)	0.43	0.28–0.66		0.44	0.28–0.69	< 0.001
Nkwanta South	51 (15.2)	0.45	0.29–0.68		0.48	0.31–0.75	0.001
South Tongu	61 (29.5)	1.03	0.67–1.59		1.11	0.71–1.72	0.65
Kadjebi	45 (13.9)	0.40	0.26–0.62		0.41	0.26–0.64	< 0.001
*Year of registration*				0.29			
2013	126 (16.9)	1					
2014	119 (15.6)	0.91	0.69–1.91				
2015	119 (17.0)	1.00	0.76–1.32				
2016	119 (18.1)	1.08	0.82–1.43				
2017	146 (19.8)	1.21	0.93–1.57				

^§^missing value: 2; *COR* Crude odds ratio; *AOR* Adjusted odds ratio, *EP* Extrapulmonary, *PP* Pulmonary positive, *PN* Pulmonary negative

## Discussion

### Case notification rate and trends

The WHO has noted that the disease burden attributed to TB is falling in most countries, but not fast enough to reach the first milestones of the End TB Strategy. The TB incidence rate per 100,000 population, therefore, needs to be falling at about 5% per year to achieve the set target by 2020 [2]. This study has demonstrated an insignificant decline in the notification rate of all forms of TB, with an average decline rate of 8.8% per year. The rate of decline is however far higher than the 1.5% global rate in the TB incidence per year during 2000–2017 and 4% in the WHO African Regions during the same period [2]. This could imply that the Volta region is on course to achieving the milestone for a reduction in TB cases by 10% per year by 2025, as set in the End TB strategy [10]. According to the Ghana health services, TB case notification in the country has declined in the last 5 years in all regions except Western and Brong Ahafo Regions [11]. However, the decline could either be due to a true reduction of the disease burden in the population or as a result of low case detection due to inadequate diagnostic facilities, weak surveillance systems, inadequate knowledge about the disease, and stigma at the community level [12], which needs further investigation. Other African countries have reported varied trends in TB case notification over the years. For instance, while Damemew et al. [13] and Senedu et al. [14] observed a downward trend in CNR of all forms of TB from the years 2011–2015 in Ethiopia, Shallo and colleague [15] noticed a consistent rise in case notification of TB during 2007–2011 in 25 districts of Arsi zone of Central Ethiopia. In Papua New Guinea, Aia et al. [16] discovered an increasing trend of TB case notification during 2008–2016 and a stabilized trend during 2015–2016. These variations in the trends of TB CNR could be as a result of differences in the period of study or differences in geographical location.

The overall CNR of all forms of TB was estimated to be 65 cases per 100,000 population, slightly higher than the Volta Regional average of 60 cases per 100,000 population in 2016 [17]. The estimated rate is however twice lower than the 2017 estimate of 133 cases per 100,000 Ghanaian population [2]. Wide variation of TB case notification rate observed among the districts studied and that of the country’s average could be a function of clinical diagnostic abilities, health care access and quality of the district surveillance system to capture cases rather than a true difference in the burden of TB by place. This, however, remains to be explored whether the variability in CNR at the district level reflects the true differences in disease burden, differences in case detection and reporting, or both.

### TB treatment outcomes

Diagnosis and successful treatment of people with TB avert millions of deaths each year that are due to the disease. However, TB treatment programs in most developing countries have not been effective as expected and hence there exist huge and persistent gaps in detection and treatment [2]. Assessment of TB treatment outcomes and the predictors for poor treatment outcomes is one of the major indicators for the evaluation of the performance of any TB control program. Contrary to previous studies in Ghana [5], and Ethiopia [18], our results point to a declined performance of treatment outcomes in 10 districts of the Volta Region during 2012-2016 treatment period. Generally, while treatment success and cure rates declined, the proportion of people who died due to TB, experienced treatment failure, and those lost to follow up showed fluctuating trends over the years but with slight increases from 2015 to 2016. This poor performance could be due to patients’ delay in seeking TB care as a result of stigma, lack of knowledge regarding TB signs and symptoms, inadequate community involvement in TB care, and lack of treatment supervision [19, 20]. Community TB care, an intervention that recognises social capital and connectedness as a tool to effective TB care and prevention has been reported to be effective in improving TB treatment outcomes in many parts of the world, including Ghana [5, 21, 22], hence needs to be pursued vigorously in the Volta region in order to achieve improved treatment outcomes. The study further revealed that about 8 in 10 of a cohort of patients with TB treated during the period had a successful treatment outcome. This result is lower than Ghana’s average (85%) [2], and rates reported in previous studies in Ghana (87.7%) [5], Iran (83.1%) [23], and a pooled analysis in Ethiopia [24]. Our finding is however consistent with other previous studies in Kenya (82.4%) [25], and South Africa (82.2%) [26], and the global average of 82% in 2017 estimated by the WHO [2]. Variations in treatment outcomes were noticed among districts, with South Tongu and Central Tongu having the poorest treatment success rates of 70.5 and 71.3% respectively as a result of high case fatality rates (20.7%) and (21.5%). Keta, Kpando, Kadjebi, Nkwanta South, Krachi East, and Krachi West had treatment success rates above the study’s average. The reason for the variations in treatment success rate could be due to differences in programme management and TB/HIV co-infection since comorbidity has been reported to have a negative influence on TB treatment [27]. TB Treatment success rate of ≥90% is required by control programmes in order to achieve the End TB Strategy targets by 2025 [11]. Coordinated efforts among tuberculosis control programs and health care professionals in assessing patient barriers to adherence and the use of innovations and ‘enablers’ that can assist the patient in adhering to the recommended therapy are fundamental requirements for achieving the milestones and targets of the End TB Strategy [2, 28]. Essentially, poor treatment adherence, which can lead to treatment failure, relapse, and the emergence of drug-resistant strains is one of the obstacles in controlling the TB epidemic [29, 30]. In this study, lost to follow up as an unsuccessful treatment outcome is comparatively lower than the findings of Kefale and Anagaw [31] and Gebrezgabiher and colleagues [32], in southwest Ethiopia who reported lost to follow up rate of 10.6 and 11.1% respectively. TB treatment interruption is an important public health problem. Patients who interrupt treatment may continue to transmit the infection to healthy people or acquire drug-resistance TB strains and consequently treatment failure [33].

TB mortality is a key indicator for monitoring progress towards the 2020 and 2025 milestones. In this study, the proportion of patients with TB who died due to the disease (13.5%) though lower than the global average (16%), a case fatality rate (CFR) of less than 6% is required to achieve the 2025 global milestone for reductions in TB deaths [2]. Our estimated CFR is consistent with a similar study in Accra, Ghana (12.6%) [34], but higher than what was found in Southern Ethiopia (3.4%) [32]. The high mortality rate due to TB could be attributed to patients’ delay in seeking treatment after symptoms have developed, as a result of poor patients’ knowledge and awareness of TB symptoms and the need for prompt consultation with healthcare services for diagnosis and treatment [19]. Contrary to the global trend [2], CFR in this study increased by about 58% over the period, a worrying phenomenon that calls for a coordinated effort by all stakeholders to reverse. We found a varied degree of CFR among districts, with the highest rates recorded in Central Tongu (21.5%) and South Tongu (20.7%) districts and lowers in Krachi East (3.1%) and Krachi West (5.1%). Other previous studies have reported a varying degree of TB death across geographical location. For instance Malede et, al [35]. in their study in Northeast Ethiopia reported 8.1% death rate, Ukwaja, Alobu, Ifebunandu, and Osakwe [36] in Southeastern Nigeria reported 5% deaths between 1999 and 2008, Gebrezgabiher, et al. [32] in Southern Ethiopia reported 3.4% between 2008 and 2013. This variation could be attributed to differences in TB care in different areas and improvement of care over the years.

Treatment failure as an unsuccessful treatment outcome is a major threat to the control of TB as these patients are at higher risk of developing drug-resistant resistant TB and often associated with poor retreatment outcomes [37]. The treatment failure rate among bacteriologically confirmed TB cases in this study though low, need to further reduce since these patients are likely to develop multi-drug resistant strains if not managed well.

### Predictors of unsuccessful treatment outcomes

Ascertaining the factors that predict unsuccessful treatment outcomes is essential to evaluating the performance of TB control programs and to design effective interventions [38]. Studies conducted across different populations identified various factors that could affect treatment outcomes of patients with TB [39, 40]. Consistent with previous studies [25, 27, 40–42] in South Africa [43] and Ghana [26], we found in this study that the occurrence of unsuccessful treatment outcomes was 1.94-fold higher among TB and HIV co-infected patients compared to patients with only TB. The unsuccessful treatment outcomes among TB/HIV co-infected patients in this study could be due to drug interactions between rifamycins (rifampin or rifabutin) and some antiretroviral agents [44] as well as malabsorption of anti-TB drugs among patients with advanced HIV [45], leading to low serum concentrations of drugs and hence unsuccessful treatment outcomes [46]. A similar study reported that TB/HIV co-infections without the use of Anti-Retroviral Therapy (ART) was associated with poor treatment outcome [47].

Unsuccessful prior TB treatment has a significant impact on subsequent treatment outcomes, with patients who returned to treatment after a prior interruption, who failed prior treatment, and those who relapsed doing poorly. In this study, patients who defaulted prior treatment had 3.62 higher odds of failing subsequent treatment compared to new patients. Poor treatment outcomes among these patients could mainly be due to mycobacterial resistance as a result of having been exposed to incomplete treatment [33, 48]. Due to serious consequences of TB treatment interruption, pragmatic efforts that ensure treatment compliance and completion are required to minimize the chances of having unsuccessful treatment outcomes and subsequent development of drug resistance. Understanding the factors that prevent patients from adhering to treatment can help minimize default.

Unlike previous studies which found old age as a risk factor for unsuccessful treatment outcome [35], factors, such as sex, age, and type of patients in this current study were not predictors of unsuccessful treatment outcomes.

This study is the first research in Ghana comparing TB programme performance of several BMCs. This provides a broad perspective of TB performance in a particular region, which may facilitate the identification of non-performing districts for better allocation of resources. Additionally, analysis of trends of TB CNR is rare in Ghana. Trend analysis of TB CNR helps to monitor the progress of achieving the programme’s targets of reducing TB incidence by 2030.

Although we believe our report presents significant lessons relevant for programme planning and implementation, it is important to acknowledge the following limitations. Firstly, the information used for the analysis was derived from routine data with potential for errors in recording, and between observer variability that is inherent in the collection and entry. This could lead to the observation of spurious results. However, the routine application of thorough checks in the registers by district and regional TB coordinators reduce the probability of these errors. Double data entry was independently done and compared by two of the co-authors with differences validated and corrected with the primary source to reduce errors. Secondly, treatment outcomes of nearly 4% of patient were not known and hence were excluded from the estimation of treatment outcomes. This could have biased our estimates of treatment outcomes and risk, resulting in under or overestimation.

## Conclusion

This analysis demonstrates an unimproved TB case notification and treatment success rates in 10 districts of Volta Region, Ghana during 2013–2017, with wide inequalities among districts. The study indicates that nearly 2 in 10 TB patients are unsuccessfully treatment, which is strongly influenced by returned after treatment interruption and co-morbidity with HIV infection. Sustained and proven interventions such as community TB DOTS, provision of “enablers” to patients, and health system efficiency may impact positively on case notification and treatment outcomes to achieve the elimination goal. Contact tracing of bacteriologically positive TB patients and targeted screening among high-risk groups can improve case notification. Additionally, strengthened TB/HIV collaborative activities such as testing TB patients for HIV and treating co-infected patients with anti-retroviral is required in order to improve TB treatment outcomes among co-infected patients.

## Data Availability

The datasets used during the current study are available from the corresponding author on reasonable request.

## Abbreviations

AIDS: Acquired Immunodeficiency Syndrome
BMU: Basic Management Unit
CNR: case Notification Rate
DOTS: Directly Observed Treatment Short-course
HIV: Human Immunodeficiency Virus
NTBCP: National Tuberculosis Control Programme
PLHIV: People Living with HIV
TB: Tuberculosis
WHO: World Health Organization
